# Can We Predict Skeletal Lesion on Bone Scan Based on Quantitative PSMA PET/CT Features?

**DOI:** 10.3390/cancers15225471

**Published:** 2023-11-19

**Authors:** Riccardo Laudicella, Matteo Bauckneht, Alexander Maurer, Jakob Heimer, Antonio G. Gennari, Tania Di Raimondo, Gaetano Paone, Marco Cuzzocrea, Michael Messerli, Daniel Eberli, Irene A. Burger

**Affiliations:** 1 Department of Nuclear Medicine, Cantonal Hospital Baden, 5404 Baden, Switzerland; riclaudi@hotmail.it (R.L.);; 2 Department of Nuclear Medicine, University Hospital Zurich, University of Zurich, 8006 Zurich, Switzerland; 3 Nuclear Medicine Unit, Department of Biomedical and Dental Sciences and Morpho-Functional Imaging, University of Messina, 98122 Messina, Italy; 4 Nuclear Medicine, IRCCS Ospedale Policlinico San Martino, 16132 Genova, Italy; 5 Department of Health Sciences (DISSAL), University of Genova, 16126 Genova, Italy; 6 Department of Mathematics, Seminar for Statistics, ETH Zurich, 8092 Zurich, Switzerland; 7 Clinic for Nuclear Medicine and Molecular Imaging, Imaging Institute of Southern Switzerland, Ente Ospedaliero Cantonale, 6500 Bellinzona, Switzerland; 8 Department of Urology, University Hospital of Zurich, 8006 Zurich, Switzerland

**Keywords:** generalized linear mixed model, prediction, prostate cancer, staging, PSMA PET

## Abstract

**Simple Summary:**

Treating patients for metastatic prostate cancer based on the information of PSMA-PET bears the risk to “under- or overtreat” patients, given that many lesions seen only on PSMA-PET but not on conventional imaging (CI) could alter their management. It is not possible to predict the disease status using CI with PSMA-PET/CT, because bone lesions can be positive on bone scintigraphy (BS), without evidence of the disease on CT. Some authors suggested using clinical parameters to predict BS results, but this does not reach enough accuracy to adjust the therapy. If an algorithm based on PSMA-PET/CT data were able to predict if lesions are visible on BS, this might be a possible way to adjust patient management based on CI-based guidelines in light of PSMA-PET/CT. Therefore, we aimed to develop a model to predict the visibility of bone lesions on BS based on PSMA-PET/CT data.

**Abstract:**

Objective: The increasing use of PSMA-PET/CT for restaging prostate cancer (PCa) leads to a patient shift from a non-metastatic situation based on conventional imaging (CI) to a metastatic situation. Since established therapeutic pathways have been designed according to CI, it is unclear how this should be translated to the PSMA-PET/CT results. This study aimed to investigate whether PSMA-PET/CT and clinical parameters could predict the visibility of PSMA-positive lesions on a bone scan (BS). Methods: In four different centers, all PCa patients with BS and PSMA-PET/CT within 6 months without any change in therapy or significant disease progression were retrospectively selected. Up to 10 non-confluent clear bone metastases were selected per PSMA-PET/CT and SUV_max_, SUV_mean_, PSMA_tot_, PSMA_vol_, density, diameter on CT, and presence of cortical erosion were collected. Clinical variables (age, PSA, Gleason Score) were also considered. Two experienced double-board physicians decided whether a bone metastasis was visible on the BS, with a consensus readout for discordant findings. For predictive performance, a random forest was fit on all available predictors, and its accuracy was assessed using 10-fold cross-validation performed 10 times. Results: A total of 43 patients were identified with 222 bone lesions on PSMA-PET/CT. A total of 129 (58.1%) lesions were visible on the BS. In the univariate analysis, all PSMA-PET/CT parameters were significantly associated with the visibility on the BS (*p* < 0.001). The random forest reached a mean accuracy of 77.6% in a 10-fold cross-validation. Conclusions: These preliminary results indicate that there might be a way to predict the BS results based on PSMA-PET/CT, potentially improving the comparability between both examinations and supporting decisions for therapy selection.

## 1. Introduction

The significantly higher accuracy of prostate-specific membrane antigen (PSMA) positron emission tomography (PET)/computed tomography (CT) over conventional imaging (CI), consisting of a bone scan (BS) and a contrast-enhanced CT, for prostate cancer (PCa) was proven in a prospective randomized trial [1], increasing the confidence of clinicians towards this novel imaging modality. This led to the widespread use of this technique not only for early biochemical recurrence (BCR) but also for PCa staging and assessments of disease progression [2]. However, despite the use of PSMA PET imaging becoming the standard in many institutions, CI remains the imaging modality of choice for the response assessment [3]. This leads to heterogeneous imaging in the follow-up of PCa patients with inherent difficulties in comparing the disease status between different modalities. Furthermore, multiple therapeutic pathways are designed according to CI, and little is known of whether those algorithms are still optimized for patients staged or restaged with PSMA PET. If we follow therapy regimens for metastatic diseases, it is still unclear if we “undertreat” patients with tumors seen only on PSMA PET (but not on CI), given that they would still be considered eligible for therapy regimens for the non-metastatic disease based on CI. In fact, in a recent study on 200 patients considered non-metastatic castration-resistant PCa (nmCRPCa) on CI, Fendler et al. showed that in 196 of them (98%), PSMA PET detected metastases [4].

Furthermore, innovative therapeutic approaches for PCa are emerging such as targeted radiotherapy (RT) for oligometastatic disease and also in patients after progression to androgen deprivation therapy (ADT) [5]. This opportunity could be withheld for the patient if we perform only CI after the start of systemic treatment.

Unfortunately, it is not possible to compare the CI-based disease status with PSMA PET/CT. Indeed, nodal or visceral diseases seen on PSMA PET/CT can be projected to the theoretical results of CT, given that the anatomic size of a lesion is assessable using both techniques. On the other hand, bone lesions can be positive on the BS without evidence on the CT component and vice versa [6]. Numerous results showing clear superior results for PSMA PET in the detection of bone metastasis [7,8], also versus SPECT/CT [9,10], underline the difficulties in the interpretation of PSMA PET/CT-positive bone lesions in comparison to BSs. Some authors suggested using clinical parameters to predict a visible lesion on BS in nmCRPCa patients [11]. However, to overrule positive bone disease on PSMA PET/CT, based on a combination of clinical information (age, Gleason Score, and PSA) only, seems not to be very convincing. Differently, if an algorithm based on PSMA PET/CT visual and quantitative data able to predict which lesion will be visible on the BS could be established, this might be the basis for better treating patients with specifically approved drugs or enhancing the ability to compare heterogeneous imaging within a patient. Therefore, this study aimed to develop a model to predict the visibility of bone lesions on a BS based on data from PSMA PET/CT.

## 2. Methods

### 2.1. Patients

**Figure 1:**
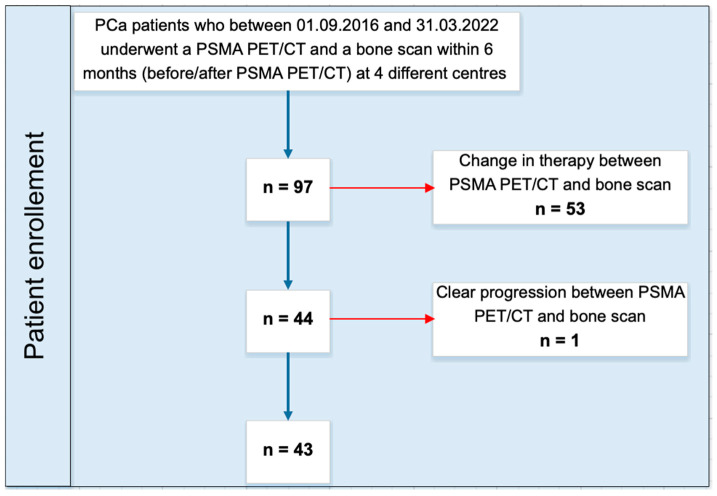
Patients’ inclusion workflow.

In this retrospective multicentric study (n = 4), we screened all PCa patients who between 1 September 2016 and 31 March 2022 underwent a PSMA PET/CT showing at least one suspicious bone lesion and a BS followed/preceded within 6 months (n = 97). Then, we included only patients without any change in therapy between both scans (n = 44), to exclude any morphologic or metabolic changes due to therapy. Finally, we excluded one case for a clear progression between the 2 scans (n = 43, Figure 1). The study was approved by the institutional review board (2021-01601) and all patients signed a general informed consent.

### 2.2. PSMA PET/CT and Bone Scan

All patients underwent PSMA PET/CT and whole-body planar BS for PCa at 4 different institutions (15/43 in-house, 28/43 at external institutions). A total of 17 patients underwent [^68^Ga]Ga-PSMA-11 PET/CT scans 60 ± 10 min after the administration of a median dose of 148.6 MBq (77–370), while 26 patients underwent [^18^F]PSMA-1007 PET/CT ninety minutes after the administration of a median dose of 319.5 MBq (210–375). Furthermore, a whole-body BS was performed 180 min after the administration of a mean dose of 717.6 ± 82.1 MBq of [^99m^Tc]Tc-HDP in 36/43 patients and of 664.9 ± 42.7 MBq of [^99m^Tc]Tc-DPD in 7/43 patients. All institutional protocols agreed with the joint EANM-SNMMI procedure guidelines [12,13].

### 2.3. Image Analysis

For each patient, a double board-certified radiologist and nuclear medicine physician centrally analyzed PSMA PET/CT images, and up to 10 PSMA PET-positive bone lesions were selected. Lesions were eligible if clearly solitary in a skeletal region (e.g., proximal humerus, fifth lumbar vertebra, and so on) to enable potential accurate co-localization on the BS. Confluent lesions were excluded. On PSMA PET/CT, a region of interest (ROI) was inserted for each lesion acquiring quantitative image parameters. On PSMA PET, we quantified PSMA uptake with SUV_max_, SUV_mean_, maximum uptake diameter (cm, with a window color scale of 0–8), and volume-based measures using a fixed threshold at SUV > 4 to delineate the total PSMA uptake (PSMA_tot_, g/mL) [14,15,16] and PSMA volume (PSMA_vol_, cm^3^). On low-dose co-registered CT, we assessed the average density in Hounsfield Units (HU), the maximum diameter if the lesion was seen on CT (cm), and the presence/absence of cortical erosion. For lesions without any morphologic changes (sclerotic or lytic) visible on CT, the mean HU was assessed in the region with the higher PSMA accumulation without incorporating the regular cortical bone, and the CT diameter was set to 0 cm. Lesions with mean HU values above 800 were considered clearly sclerotic, given that regular spongy bone should range between 200 and 700 HU in adults [17].

With the list of up to ten regions per patient, two double-boarded radiologists and nuclear medicine physicians blinded for the PSMA PET/CT data assessed the clinical information (indication for BS, PSA value, age) and regular planar BS in anteroposterior and posteroanterior projection in two windows. For each region, each reader decided whether a suspicious lesion was present; for discrepant results, a consensus read was performed and considered as the standard of reference for the presence of skeletal lesions in the BS.

PSMA PET/CT images were assessed by one nuclear medicine physician with 3 years of experience. BS images were assessed by two dual-boarded radiologists and nuclear medicine physicians with 9 and 11 years of experience, respectively.

All images were centrally analyzed in a dedicated review workstation (Advantage Workstation, Version 4.7, GE Healthcare), which enables the review of the PET and CT images side by side and in fused mode.

**Figure 2:**
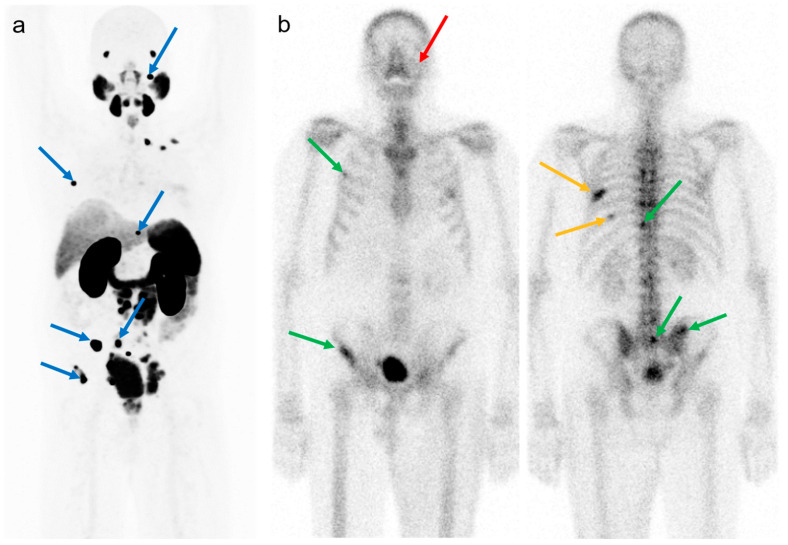
Example of comparison between [^68^Ga]Ga-PSMA-11 PET/CT and BS. (**a**) Coronal maximum intensity projection (MIP) of [^68^Ga]Ga-PSMA-11 PET/CT and (**b**) coronal projections of a BS with [^99m^Tc]Tc-DPD acquired 3 weeks after the PSMA PET. Lesions were selected on PSMA PET and labeled from cranial to caudal: the left mastoid, 3rd right rib, Th10, sacrum, right sacroiliac joint, and right iliac wing (blue arrows). All lesions but the left mastoid (red arrow) were labeled positive on the BS (green arrow). Of note, the intense uptake in the 7th and 9th ribs (orange arrow) on the left had corresponding fracture lines on PSMA PET with only minimal uptake.

In Figure 2, we describe an example of the comparison between [^68^Ga]Ga-PSMA-11 PET/CT and BS.

### 2.4. Statistical Analysis

Descriptive analyses were used to display patient data as means with standard deviations or medians with ranges to describe normally or non-normally distributed values, respectively; a frequency distribution with percentages was used to summarize categorical variables. Univariate differences in the means of the imaging variables (PSMA SUV_max_, SUV_mean_, PSMA_tot_, PSMA_vol_, maximum uptake diameter, maximum lesion diameter at CT, HU) and the clinical variables (age, International Society of Urological pathology—ISUP—grade, PSA at PSMA PET/CT, days between scans, PSA change between scans, and lesion location) between the positive and negative lesion visible on thet BS were assessed with t-tests. Chi-square tests of independence were used for the assessment of dependence between the lesion visibility on the BS and the presence of cortical erosions, the applied tracer, the lesion location, and the ISUP grade. To evaluate the interrater agreement on BS assessment, Cohen’s kappa test was used. Values < 0.2, 0.21–0.39, 0.4–0.59, 0.6–0.79, 0.8–0.9, and >0.9 were considered of none, minimal, weak, moderate, strong, and almost perfect agreement/reliability, respectively [18]. Missing values (ISUP: 3, PSA: 1) in the cohort were imputed by respective mean values.

For the inference study, a linear mixed logistic regression (generalized linear mixed model—GLMM) was fit to all 8 imaging variables and the 6 abovementioned clinical variables as fixed effects, and random intercepts were used for the patient identifier and the study center. The model was fit with the package lme4 [19]. Then, backward elimination was performed with the package buildmer [20]. Pseudo-R-squared for generalized mixed-effect models was computed with the package MuMin [21].

For the prediction study, a random forest was fit to all 8 imaging and 6 clinical variables. A 10-fold cross-validation was performed 10 times. The reported accuracy of lesion visibility represents the mean of all runs and all folds. The patient identifier and the study center were not used in the prediction study. For the comparisons of the random forest model with a simple binary regression using only the visibility on CT (lesion’s CT diameter > 0 cm) as a predictor, receiver operator characteristics (ROC) were built and the area under the ROC-curve (AUROC) was calculated and compared with Delong’s test.

A *p*-value of less than 0.05 was considered statistically significant. Statistical analyses were performed with R [22] and SPSS (IBM). Analyses were performed by JH (MD and statistician), RL, and IAB (nuclear medicine physicians).

## 3. Results

We identified forty-three patients (mean age at first scan 73.2 ± 8.5 years; median PSA at first scan 13.15 ng/mL, 0.34–2189) who underwent PSMA PET/CT and a BS within a mean of 55 ± 49.3 days. Most of the patients were scanned for CRPCa (74.5%). The initial mean ISUP grade was four. The main patient characteristics are given in Table 1.

On PSMA PET/CT scans, 222 lesions were selected. The lesions were distributed as follows: ribs 42/222 (19%), spine 69/222 (31%), pelvis 44/222 (20%), extremities 50/222 (22.5%), sternum 7/222 (3%), and skull 10/222 (4.5%).

There was a wide range of PSMA activity for SUV_max_ (1.8–95) as well as PSMA_vol_ (0–103 cm^3^). For lesions with SUV_max_ < 4, the PSMA volume parameters were considered = 0 cm^3^ (n = 11). CT diameter was non-measurable and set to 0 cm for 53 lesions without any morphologic correlate on CT (considered invisible on CT); of these, 19 (35%) lesions were positive on the BS.

On the other hand, measurable lesions on CT with clear sclerosis (HU > 800, n = 39) were negative on the BS in 14/39 (36%). Lesion characteristics are given in Table 2.

In total, 129 of 222 (58%) PSMA-positive skeletal lesions were visible also on the BS, according to the consensus read out. The inter-rater agreement between the two double-board radiologists and nuclear medicine physicians was moderate with k = 0.653.

### 3.1. Visible Versus Non-Visible Lesions on BS

#### 3.1.1. Univariate Analysis

**Figure 3:**
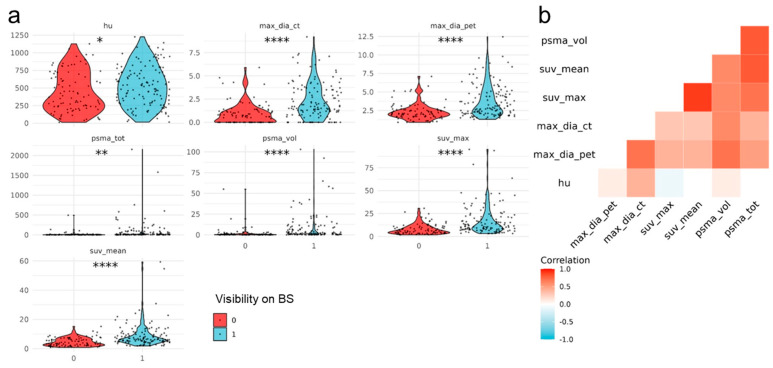
(**a**) Violin plots for the imaging parameters on PSMA PET/CT that had a significant correlation with the lesion visibility (1 = visible, 0 = not visible) on BS: bone density (HU), maximum diameter on CT, maximum diameter on PET, PSMA_tot_, PSMA_vol_, SUV_max_ and SUV_mean_ with * representing the significance of the t test (* < 0.05, ** < 0.01, **** < 0.0001). (**b**) Illustration of the correlation between the 6 parameters that were significant in univariate analysis, indicating the high amount of shared information between the imaging parameters.

SUV_max_, SUV_mean_, PSMA_vol_, PSMA_tot_, and max diameter on PET (uptake) and CT were significantly higher in visible (129/222) compared to non-visible BS lesions (93/129) (*p* < 0.001) as shown in Table 3. The strongest association with lesion visibility was observed for the max diameter on CT and SUV_max_ from PSMA PET. In addition, the lesion density on CT (HU) was significantly higher in BS-positive lesions compared to negative ones (*p* = 0.018). The slight difference in terms of the presence of cortical erosion between the two groups did not reach significance (*p* = 0.064); no significant difference between groups was also observed for PSA (*p* = 0.46) and ISUP values (*p* = 0.71, Figure 3).

#### 3.1.2. Inference Study

The variable importance of the mixed-effects logistic regression fit to the 8 PSMA PET/CT parameters and 6 clinical variables (age, ISUP, PSA at PSMA PET/CT, days between scans, PSA change between scans, and lesion location) and random intercepts for the patient identifier and the study center were analyzed by a backwards elimination procedure. This procedure stopped with the variables SUV_max_ and maximum diameter on CT still included in the model and an Akaike information criterion (AIC) of 244.4. The final model had a pseudo marginal R2 of 0.38 and a pseudo conditional R2 of 0.54.

#### 3.1.3. Prediction Study

**Figure 4:**
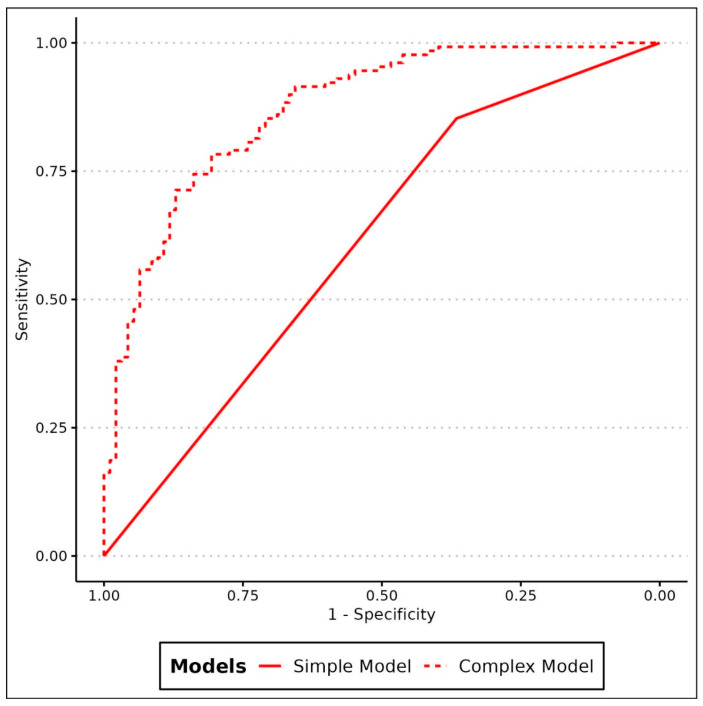
Comparison of the random forest model (complex model) based on 8 imaging parameters (dotted line) yielding an AUROCC of 0.82. In comparison to a simple model just considering the visibility on CT reaching an AUROC of 0.61.

A random forest was fit to the 8 imaging and 6 clinical variables. In the 10-times repeated 10-fold cross-validation, the random forest classifier attained a mean accuracy over all the runs and folds of 77.6%. The corresponding AUROC of the random forest was 0.82, which was significantly higher compared to the AUROC of a simple binary regression with only CT-visibility as a predictor (0.61, *p* < 0.001, Figure 4).

**Figure 5:**
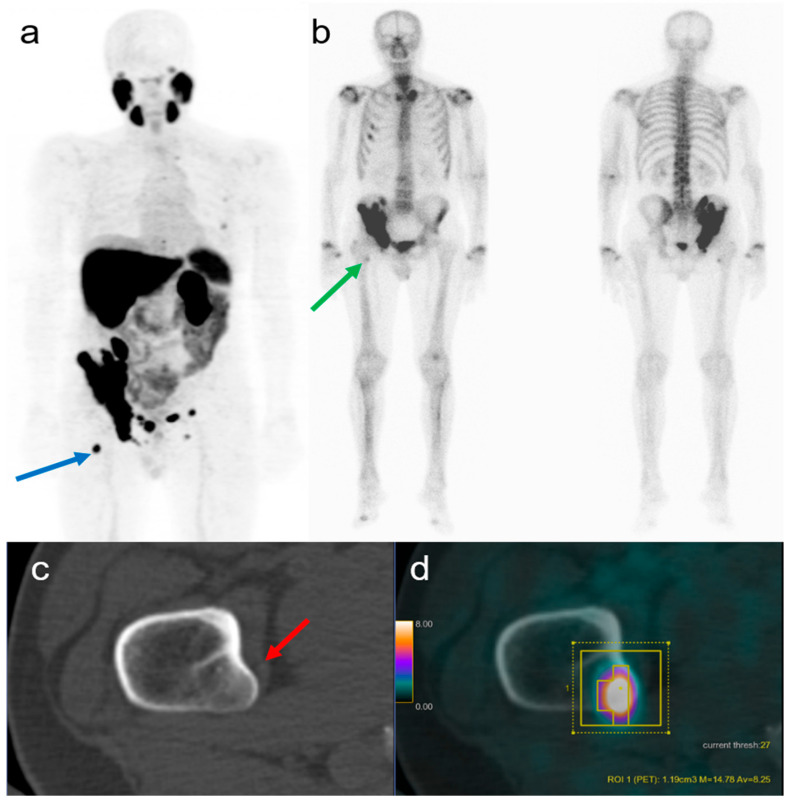
(**a**) Coronal MIP of a [^68^Ga]Ga-PSMA-11 PET/CT, with a selected lesion in the right proximal femur (blue arrow). (**b**) Coronal projections of a BS with [^99m^Tc]Tc-DPD acquired 7 weeks before the PSMA PET with increased uptake in the right proximal femur rated as visible by both readers (green arrow). (**c**) Axial CT scan in the bone window, not showing clear sclerosis or measurable correlate for the lesion (red arrow). (**d**) Axial [^68^Ga]Ga-PSMA-11 PET/CT showing intense PSMA uptake, with a SUV_max_ of 14.8, SUV_mean_ 8.25 and PSMA_vol_ 1.2 cm^3^; based on the random forest model, the lesion was characterized as visible on BS, despite the absence of evident morphological alterations on the CT component.

Figure 5 illustrates a PSMA-positive bone lesion without any clear morphologic correlate on CT that was well seen on the BS and suggested to be positive according to the random forest.

## 4. Discussion

Despite a very heterogeneous cohort and a relatively long-time interval between BS and PSMA PET/CT in the current data set, the random forest model with 10-fold cross-validation yielded an accuracy of 77.6% to predict the visibility of PSMA-positive bone lesions on the BS. This is superior compared to a model that always assumes visibility on the BS (accuracy of 58.1%, namely the percentage of visible lesions). The accuracy was also significantly higher for the random forest model based on PET/CT parameters compared to a simple model incorporating visibility on CT alone (AUROC 0.82 vs. 0.61, *p* < 0.001).

The presence of a simple and linear relationship between PSMA PET and the bone scan was not expected due to the different uptake mechanism for the “oncotropic” PSMA able to detect PSMA-overexpressed cells and the “osteotropic” HDP/DPD able to detect areas of increased osteoblastic activity.

Backward elimination ended with only two continuous variables, indicating a strong correlation between CT and PET variables (Figure 3b). Despite the univariate significance of the t-tests, the shared information on lesion visibility appears to be high between PSMA PET and CT parameters. Due to the shared information among the selected imaging predictors, there was not a higher predictive accuracy attainable, despite the univariate significance of the mean comparisons of all parameters between visible and non-visible lesions.

The fact that a larger size is related to visibility on the BS is not surprising. Given that more aggressive tumors tend to have higher PSMA expression [23,24], the increased visibility on the BS of lesions with higher PSMA SUV_max_ seems plausible. Differently, it might not be very intuitive that the density on CT (HU) of bone lesions did not remain significant. This might be explained by the fact that visibility on CT alone does not give a good answer if a lesion is present on the BS or not, given that 35% of the lesions negative on CT were positive on the BS, while 36% of measurable lesions on CT were negative on the BS. Therefore, the proposed complex model incorporating PET and CT data yielded better results compared to the simple “visibility on CT” to predict a visible lesion on the BS (Figure 4). Figure 5 is a nice example showing the difficulty of using PSMA PET/CT information to categorize patients into high- or low-volume diseases as according to the CHAARTED criteria [25]. The lesion in the proximal right femur is relatively small and not sclerotic, based on the intense PSMA-uptake however, it is very active. The proposed model would suggest that the lesions is visible on a bone scan, correctly assigning the high-volume category for this patient.

The inclusion of both [^18^F]PSMA-1007 and [^68^Ga]Ga-PSMA-11 in this study reproduces a real-life scenario but can represent a limitation for a direct comparison of SUV values in bone lesions. Indeed, it has been shown that [^18^F]PSMA-1007 has a higher accumulation in bone metastasis compared to [^68^Ga]Ga-PSMA-11 [26]. Furthermore, we know that bone marrow mild-to-moderate uptake on [^18^F]PSMA-1007 with no morphologic correlate on CT (the so-called unspecific bone uptake lesions [27]) can be a false positive [28], even if initial studies assessing SUV cutoffs for malignancy are increasing in the literature [6,29]. Similarly, solitary [^68^Ga]Ga-PSMA-11 PSMA-avid lesions in the ribs should be interpreted cautiously as they may represent false-positive findings [8]. Furthermore, we collected data from four different centers with different scanners and reconstruction algorithms, again limiting the absolute comparability of SUV values. However, despite this heterogeneity, the proposed model based on quantitative PET parameters achieved a higher AUC (0.825) compared to the simple use of visibility on CT (0.61).

Bone scans are not commonly used for staging anymore, but still play a pivotal role in the response assessment given that PSMA PET/CT is not an approved modality for this yet and is recommended to be used only within clinical trials [30]. These trials are currently ongoing (NCT03767244 (PROTEUS), NCT05794906 (ARASTEP)), but results are still lacking. Furthermore, bone scans are also still pivotal for patient categorization into low- and high-volume diseases based on the CHAARTED criteria [25,31]. Considering the magnitude of the stage migration phenomenon from conventional imaging to PSMA PET/CT [32,33,34], a translation between PSMA PET/CT and bone scan findings would be of tremendous help to prevent the systematic up-staging to high-volume disease based on PSMA PET/CT findings.

An inherent limitation of studies assessing bone metastasis is the lack of a histopathological gold standard. Ethical considerations regarding additional pain and inherent difficulties in performing PET/CT-guided biopsies would not justify a large enough cohort with biopsy-proven metastasis. Therefore, we decided to strictly focus on image comparability, regardless of the nature of the lesion. Furthermore, the moderate kappa for the read out of the BS between two expert readers is limiting as well. The insecure labeling of the gold standard limits the potential of every model, which is a general problem for predictions of subjective parameters such as lesion visibility. Finally, in our population, we have to point out the presence of a certain heterogeneity in terms of the main ongoing therapy which may hamper PSMA expression (i.e., short term ADT can increase PSMA expression, while long-term ADT may reduce PSMA expression).

## 5. Conclusions

These preliminary results indicate that there might be a way to predict BS results based on PSMA PET/CT. This could improve the comparability between both examinations and help to interpret PSMA PET/CT results in the light of the CI-based guidelines for accurate therapy selection in patients with metastatic PCa in the future. The currently achieved accuracy is insufficient for reliable predictions, but it might be improved in larger cohorts or with the integration of further features that need more standardization (i.e., radiomic).

## Figures and Tables

**Table 1:** Patients’ main characteristics.

**Patients’ Number**	**43**
**[^68^Ga]Ga-PSMA-11—[^18^F]PSMA-1007**	17–26
**Mean age at first scan** **± SD**	73.2 ± 8.5 years
**Median PSA at first scan** (n = 42)	13.15 ng/mL (0.34–2189)
**Mean days between PSMA PET and bone scan**	55 ± 49.3 days
**Stage of disease**	
Staging	5/43 (11.5%)
HSPCa	3/43 (7%)
EBR	3/43 (7%)
CRPCa	32/43 (74.5%)
**Ongoing main therapy**	
None	10/43
ADT	10/43
Enzalutamide	3/43
Chemotherapy	2/43
223Ra	4/43
Leuprorelin	2/43
Abiraterone	6/43
Abiraterone + Leuprorelin	2/43
ADT + Abiraterone	1/43
ADT + 223Ra	1/43
ADT + Enzalutamide	2/43

Legend: PSMA, prostate-specific membrane antigen; BS, bone scan; SD, standard deviation; PSA, prostate-specific antigen; HSPCa, hormone-sensitive prostate cancer; EBR, early biochemical recurrence; CRPCa, castration-resistant prostate cancer; ADT, androgen deprivation therapy.

**Table 2:** Lesion characteristics.

Lesion Number (BS+; BS−)	222 (129;93)
Sternum	7 (3%) (5;2)
Skull	0 (4.5%) (4;6)
Rib	42 (19%) (22;20)
Pelvis	44 (20%) (26;18)
Extremities	50 (22.5%) (35;15)
Spine	69 (31%) (37;32)

**Table 3:** Quantitative parameters for BS visible vs. non-visible lesions. Continuous variables are presented as mean ± SD; categorical variables are shown as counts and proportions.

	BS Non-Visible	BS Visible	*p*	Test
**Number of lesions**	93	129		
[^68^Ga]Ga-PSMA-11	43 (46.2%)	46 (35.7%)	0.148	Chi-Squared
[^18^F]PSMA-1007	50 (53.8%)	83 (64.3%)		
**PSMA Parameters**				
SUV_max_	7.94 (5.67)	17.08 (16.19)	**<0.001**	*t*-test
SUV_mean_	4.60 (2.78)	8.85 (8.03)	**<0.001**	*t*-test
PSMA_vol_	1.65 (6.11)	8.35 (16.44)	**<0.001**	*t*-test
PSMA_tot_	12.62 (54.05)	90.94 (258.98)	**0.005**	*t*-test
Max-diameter PET	2.32 (1.12)	3.71 (2.13)	**<0.001**	*t*-test
**CT Parameters**				
Max-diameter CT	0.93 (1.15)	2.32 (1.95)	**<0.001**	*t*-test
Density (HU)	440.90 (282.84)	532.44 (283.05)	**0.018**	*t*-test
No cortical erosion	90 (96.8)	115 (89.1)	0.064	Chi-Squared
With cortical erosion	3 (3.2)	14 (10.9)		
**Clinical Parameters** Age (y)	74.11 (6.88)	72.64 (9.86)	0.217	*t*-test
PSA (ng/mL)	216.39 (500.14)	165.85 (506.89)	0.457	*t*-test
PSA change (ng/mL)	75.89 (282.19)	29.25 (138.52)	0.487	*t*-test
Days between scans	26.21 (71.05)	27.18 (65.82)	0.917	*t*-test
ISUP 1	2 (2.2)	2 (1.5)	0.71	Chi-Squared
2	12 (12.9)	17 (13.2)		
3	3 (3.2)	10 (7.8)		
4	38 (40.9)	49 (38.0)		
5	38 (40.9)	51 (39.5)		

Legend: BS, bone scan; SUV, standardized uptake value; max maximum; PSMA_vol_ = Volume of lesion with an SUV > 4; PSMA_tot_ = total PSMA accumulation within the defined lesion volume. HU = Hounsfield units.

## Data Availability

The dataset analyzed during the current study is available from the corresponding author on reasonable request.
